# Challenge of evolving *Klebsiella pneumoniae* infection in patients on hemodialysis: from the classic strain to the carbapenem-resistant hypervirulent one

**DOI:** 10.7150/ijms.69577

**Published:** 2022-01-31

**Authors:** Shuai Zhou, GuangWei Ren, YuKun Liu, XiaoMing Liu, LiHong Zhang, ShuFeng Xu, Tao Wang

**Affiliations:** 1 Graduate School of HeBei Medical University, No.361 East Zhongshan Boulevard, ShiJiaZhuang 050011, P.R. China.; 2 Department of Nephrology, the First Hospital of HeBei Medical University, No.89 East DongGang Road, ShiJiaZhuang 050030, P.R. China.; 3 Faculty of Nursing, HuaXin College of Hebei Geology University, No.69 BeiHuanGang Road, ShiJiaZhuang 050702, P.R. China.; 4 Department of Respiratory Diseases, the First Municipal Hospital of QinHuangDao city, No.258 WenHua Road, QinHuangDao 066099, P.R. China.

**Keywords:** hypervirulent *Klebsiella pneumoniae*, carbapenem-resistant, maintenance hemodialysis, antibiotic, arteriovenous fistula

## Abstract

Loss of renal function may render hemodialysis patients more susceptible to infectious diseases, which is the second of all-causes mortality in this population. In addition to infection caused by the classic *Klebsiella pneumoniae* (cKp), however, hemodialysis staffs are now facing new challenge with growing prevalence of the carbapenem-resistant Kp (CR-Kp) and hypervirulent Kp (hvKp) as they are respectively associated with increased drug-resistance and virulence. We therefore chose to share our recent experience in treating severe infections either caused (cKp, CR-Kp, hvKp) or complicated (CR-hvKp) by these strains in hemodialysis patients. Based upon yet beyond published works, we further came up with the detection of intracranial lesion, novel diagnostic approach using unique biomarkers followed by selection of appropriate antibiotics, management of metastasic abscesses and bracing for the most lethal scenario in the order of cKp, CR-Kp, hvKp and CR-hvKp, respectively. Since reports of complicated hvKp infection in hemodialysis patients were rare, we also discussed in details this clinical entity focusing on its epidemiology, mechanism of increased virulence and involvement of the arteriovenous fistula as insidious source of persistent septicemia. By covering the full spectrum of clinically relevant Kp stains specifically from the viewpoint of nephrology, our work had highlighted the importance of infection control in uremic state and *vice versa*. As such, it may greatly raise the awareness of dialysis staffs against the challenge of evolving *Klebsiella pneumoniae* infection in hemodialysis patients and expeditiously reach a higher degree of readiness which was proved to be the key determinant of ultimate survival.

## Introduction

It is well known that loss of renal function is a proximate cause of infection such that annual mortality rate secondary to sepsis was 100- to 300-fold higher in patients on dialysis than in the general population 1. Preciously, infectious diseases were the second cause of death in patients on hemodialysis, accounting for 21.6% of all-causes mortality 2. Of note, *Klebsiella pneumoniae* (Kp) was found to be either the most or second prevalent gram-negative pathogen in the hemodialysis-associated pneumonia 3, 4. According to their distinct accessory genome, the modern Kp family was divided into the classic (cKp), hypervirulent (hvKp), carbapenem-resistant (CR-Kp) and carbapenem-resistant hypervirulent (CR-hvKp) strains 5. This part was further clarified in the section of CR-Kp infection, considering the complexity and clinical relevance of the accessory genome. The emergence and rapid spreading of the hvKp and CR-Kp in geography is particularly worrisome, especially since the Kp is a key trafficker of drug resistance genes among their cohorts and other enterobacteriaceae 6. Facing these challenges, however, hvKp infection in patients on hemodialysis has received relatively little attention to date.

Hypervirulent *Klebsiella pneumoniae* is an evolving pathotype that is more virulent than the cKp and rapidly becomes an archenemy to clinicians with a growing tendency of multidrug resistance 7. A unique feature of hvKp bacteremia is the high proportion of cases that occur with no infectious source immediately apparent. Clinical manifestations usually present as infection at multiple sites or subsequent metastatic spread including liver abscesses, pneumonia, osteomyelitis, endophthalmitis and meningitis 8. By its inherent nature, the hvKp has rendered healthy individuals more susceptible to and immunocompromised ones at greater risk for the related infections 9. Moreover, acquisition of drug-resistant genes may transform the hvKp into the deadly CR-hvKp. As such, these precarious circumstances have led to a rise in the number of severe infections and the increasing scarcity of effective treatments.

Diabetes and dialysis are known risk factors for the cKp infection. In this regard, diabetics have impaired bacterial defenses, including altered chemokine and cytokine production, neutrophil responses, and phagocytic capabilities 10. Reportedly, about half of community-acquired cKp bacteremia occurs in diabetics 9. Likewise, uremia can be detrimental to the immune system, explaining the higher prevalence of cKp infection and poor outcome in patients on maintenance hemodialysis (MHD) 11. Not surprisingly, diabetic patients on MHD were more vulnerable to the very infection 12. Nonetheless, refractory infection in this population caused by the hvKp with the involvement of the arteriovenous fistula (AVF) has not been described. We herein came up with a concise and pointed review, looking into current knowledge surrounding the scenarios elicited by the cKp, CR-Kp, hvKp and CR-hvKp in hemodialysis patients.

The study was approved by our institutional ethic committee (No. 20210208) and written informed consent was obtained from the index patients for publication of this report and any accompanying images.

### Severe infection caused by the cKp

**Figure 1:**
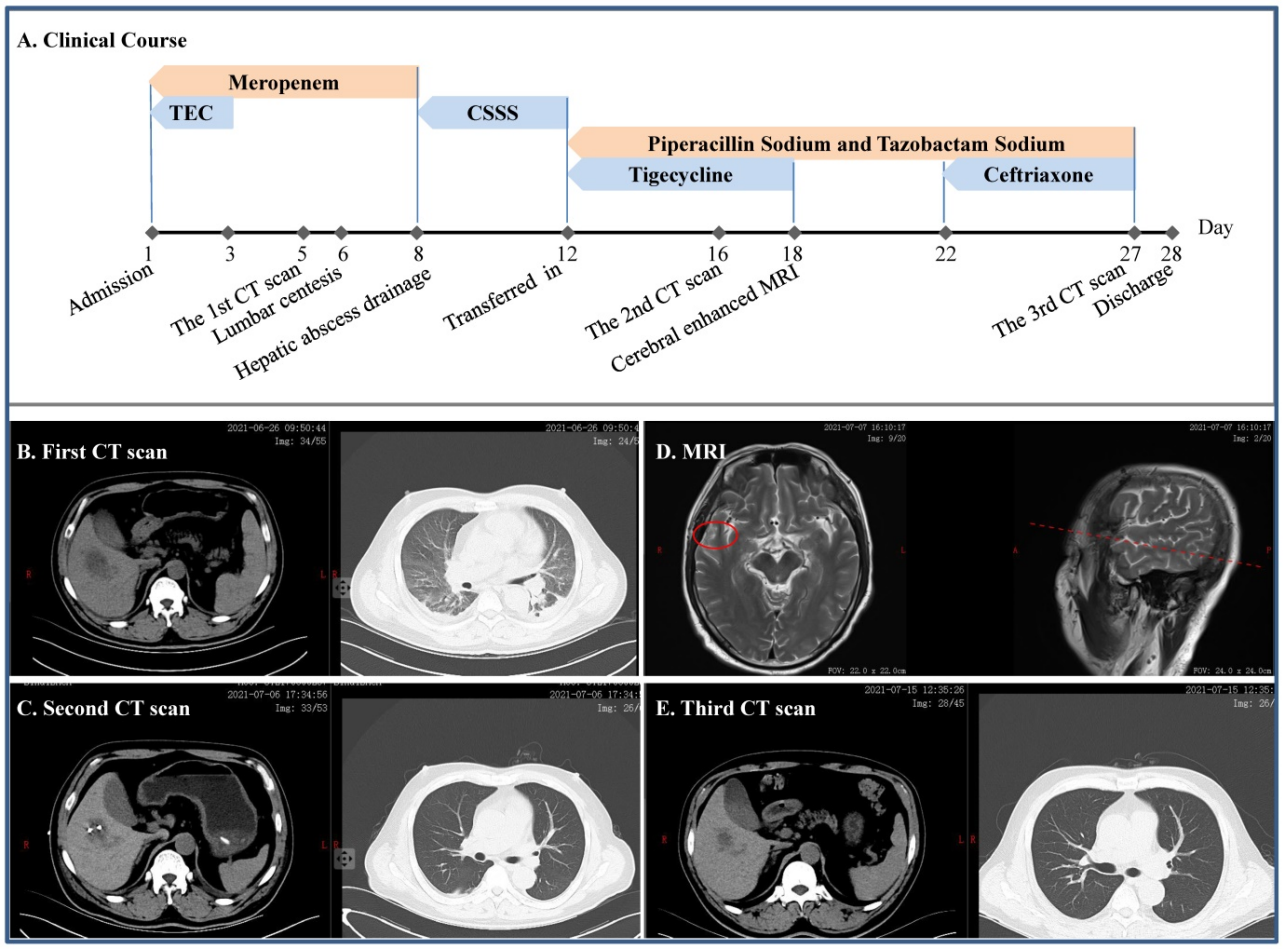
** Clinical course and imaging findings in the patient with cKp infection. A:** Major therapeutic procedures during the patient's hospital stay. **B:** The first CT scan after admission showing pulmonary infection and hepatic abscess. **C:** The second CT scan after the patient was transferred in showing gross normal pulmonary field and drained hepatic abscess. **D:** Contrast-enhanced MRI showing lesion on the right temporal lobe. **E:** CT scan prior to discharge. TEC and CSSS: teicoplanin and cefoperazone sodium and sulbactam sodium, respectively.

A 49-year-old man without self-reported diabetes was admitted to the intensive care unit (ICU) due to fever of 39.0 ºC for 2 days, accompanied by headache, disorder of consciousness and seizure (Figure 1). He was found then to have liver abscess, pulmonary infection, septic shock, multiple organs failure including respiratory failure and acute kidney injury (AKI), digestive tract bleeding, impaired coagulation and, actually, diabetes. Meropenem and tigecycline were initially given, which were replaced by cefoperazone sodium and sulbactam sodium. Mechanical ventilation and continuous renal replacement therapy (CRRT) were employed *ad interim*, lumbar centesis and hepatic abscess drainage performed, whereas cerebrospinal fluid yielded no positive finding but metagenomics next generation sequencing (mNGS) identified cKp from his blood. Once transferred to our department, we gave him piperacillin sodium and tazobactam sodium in combination with tigecycline, which was switched to ceftriaxone after a headache-driven contrast-enhanced cerebral MRI found lesion to his right temporal lobe. Free of headache and detached from hemodialysis, the patient eventually had full recovery.

Classic *Klebsiella pneumoniae* is generally an opportunistic pathogen causing infections primarily in immunocompromised individuals within the health care setting 7. In the presence of biliary disease, cKp could not only cause hepatic abscess 13, but was the commonest underlying pathogen with an isolation rate of 63.8% 14. Nonetheless, the attending nephrologists should be aware that a search for insidious diabetes mellitus is warranted in all patients with cKp hepatic abscess, as in the current case. More importantly, cKp strains originated from hepatic abscess are prone to develop meningitis 15. In this context, we believed that specific mental or physical abnormalities were essentially determined by and may in turn imply the location of brain lesion 16. Taken together, these were the rationales for our sequential selection of tigecycline and ceftriaxone in the joint antibiotic use respectively against cKp-associated hepatic abscess 17 and intracranial infection 18.

### Severe infection caused by the CR-Kp

**Figure 2:**
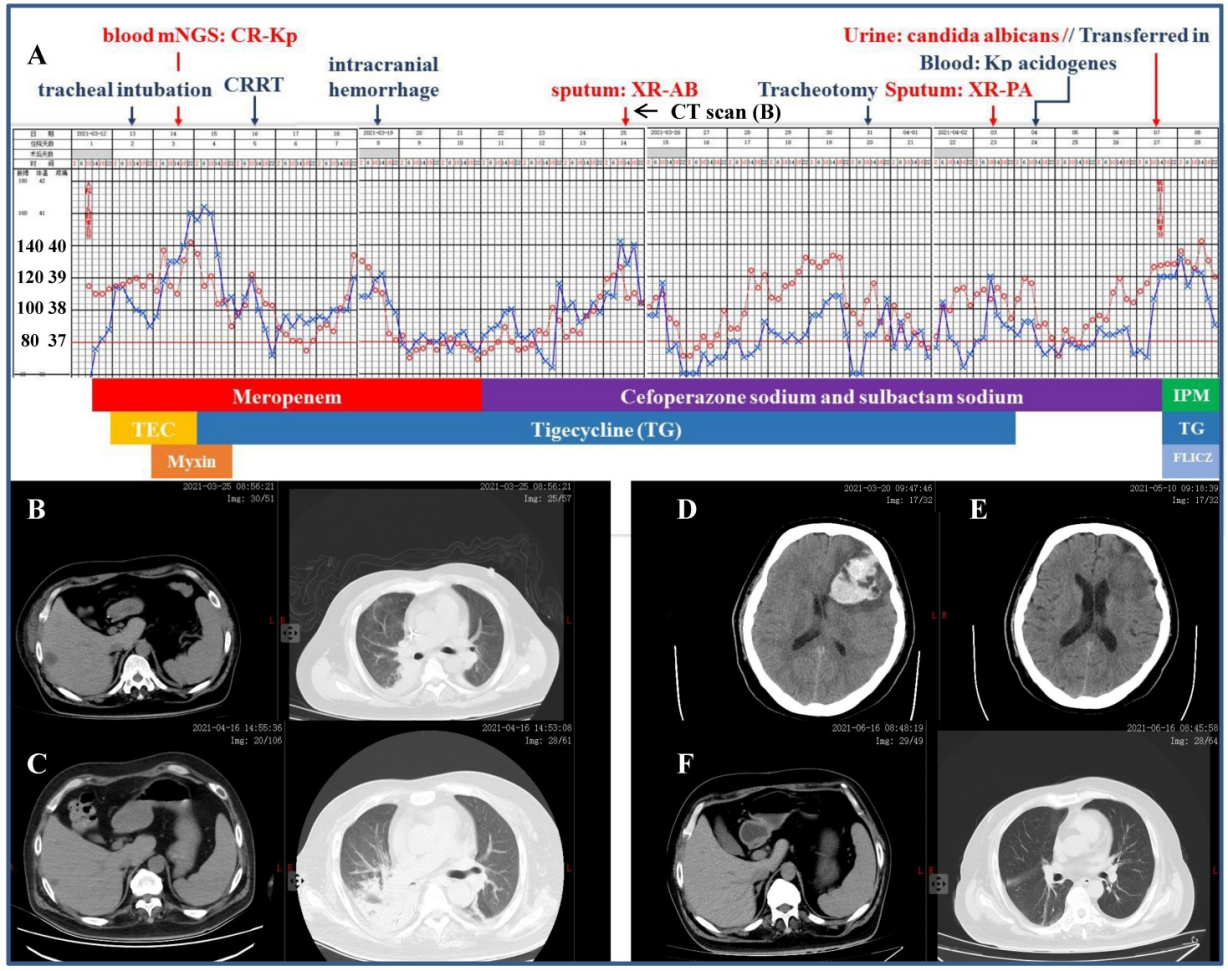

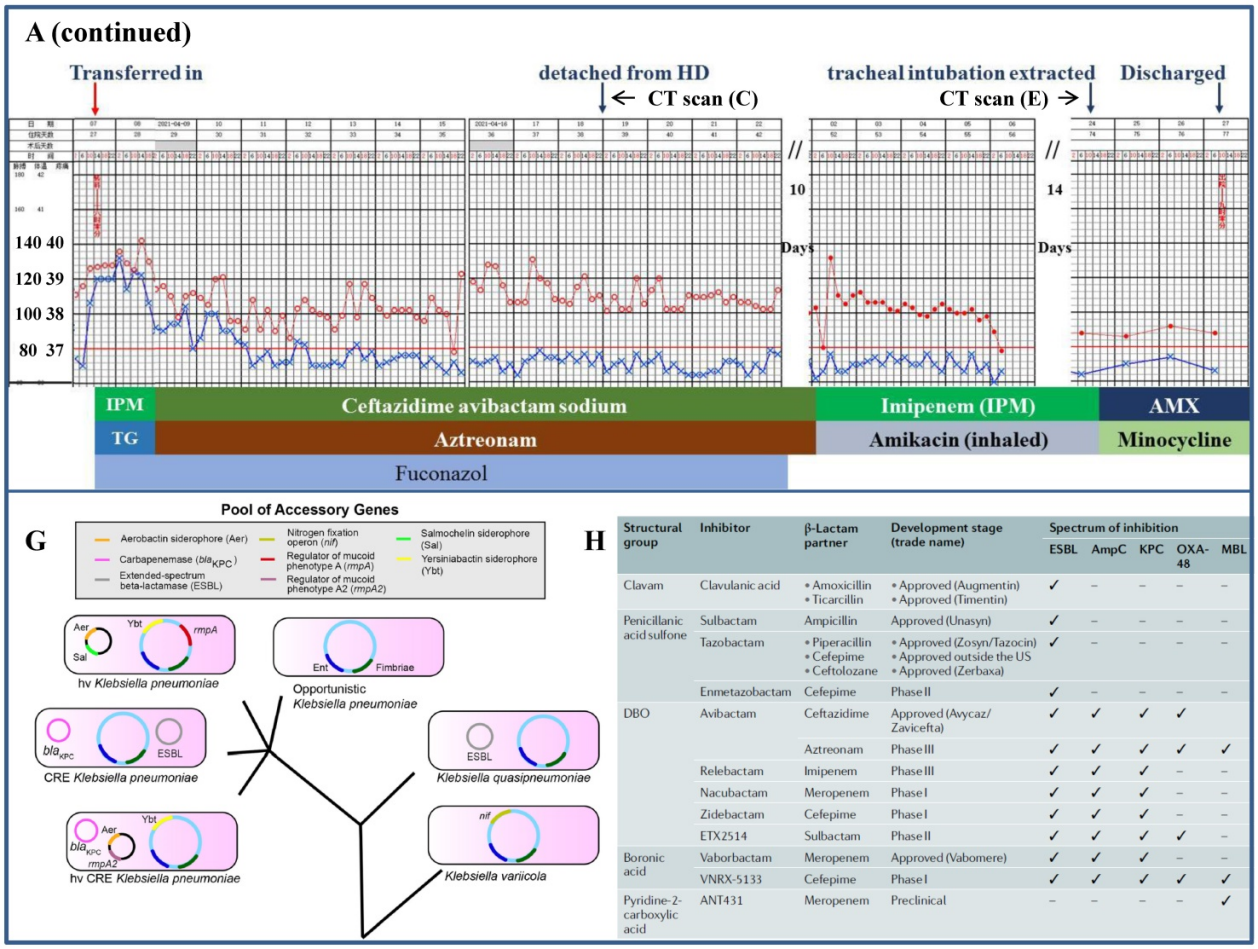
** Clinical course and imaging findings in the patient with CR-Kp infection. Part-1 Management of the patient prior to his transferring to our department. A:** Major therapeutic procedures during the patient's hospital stay. Red dots and blue cross indicated the patient's heart rate and temperature, respectively. Joint antibiotics use was at the bottom of the panel. **B:** CT scan 2-week after admission showing pulmonary infection and hepatic abscess. **C:** CT scan 12-day after the patient was transferred in showing right segmental atelectasis and significantly reduced hepatic abscess. **D and E:** CT scans immediately after the onset of cerebral hemorrhage and 2-week prior to his discharge, respectively. **F:** CT scan 10-day shortly before the discharge. **Part-2 Management of the patient after his transferring to our department.** A (continued): Major therapeutic procedures and joint antibiotics use at the bottom of the panel. G: accessory genes by which the four distinct Kp strains were genetically defined (adapted from Ref. 5 by Martin RM *et al*., Front Cell Infect Microbiol. 2018) H: comparison of the β-lactamase inhibitors and spectrum of inhibition. (adapted from Ref. 25 by Bush K *et al*., Nat Rev Microbiol. 2019) mNGS: metagenomics next generation sequencing. CRRT: continuous renal replacement therapy. XR-AB and XR-PA: multiple drug resistance Acinetobacter baumannii and Pseudomonas aeruginosa, respectively. HD: hemodialysis. TEC: teicoplanin. IPM: imipenem. FLICZ: fluconazol. DBO: diazabicyclooctanone analogue. ESBL: extended-spectrum β-lactamase. MBL: metallo-β-lactamase.

A 41-year-old diabetic man was referred to our ICU for a 2-day's history of fever pegging at 38.5 ºC and dyspnea, which was preceded by rigor (Figure 2). According to the workup on admission including CT scan which detected pulmonary infection and hepatic abscess, the initial diagnosis was septic shock, liver abscess and multiple organs dysfunction comprising of circulatory, respiratory and renal failure, type 2 diabetes and thrombocytopenia (platelet 38×10^9^/L, reference 100-300×10^9^/L). Mechanical ventilation, mNGS of the blood (with CR-Kp subsequently identified) and CRRT were initiated in short notice. The antibiotic make-up was composed of meropenem followed by cefoperazone sodium and sulbactam sodium, mainly accompanied by tigecycline. However, the patient remained febrile and his condition was further complicated by intracranial hemorrhage, isolation of multiple drug-resistant *Acinetobacter baumannii* and *Pseudomonas aeruginosa* from the sputum, Kp *acidogenes* from the blood, and *Candidas albicans* from the urine. He was then transferred to our department after a 26-day stay in the ICU, with the temperature still fluctuating around 39.0 ºC and more pronounced right segmental atelectasis. Our antibiotic regimens included three consecutive packages as following: ceftazidime avibactam sodium, aztreonam and fluconazol for 23 days, imipenem and amikacin (aerosol inhalation) for 23 days, and amoxicillin potassium clavulanate and minocycline for 3 days until discharge. Reassuringly, the patient's temperature returned to normal two days after the application of ceftazidime avibactam sodium regimen and remained so thereafter. When discharged, the patient had platelet count of 312×10^9^/L and serum creatinine of 48.7 µmol/L (reference 57.0-97.0 µmol/L), without discernible pulmonary, hepatic and intracranial lesions.

It was realized nearly two decades ago that timely nephrology consultation for patients with acute renal failure may effectively lower their mortality 19 and nowadays critical care nephrology has been an integral part of modern medical system. Without doubt, it is essential that practicing nephrologists caring for critically ill patients be more familiar with appropriate antibiotic treatment as mentioned briefly but not covered closely in the pertinent Core Curriculum 2020 20. In a coherent manner, this field of knowledge has to start from the point that Kp is highly liable to develop and transmit antibiotic resistance.

Martin *et al*. 5 clearly unraveled that Kp had a large accessory genome of plasmids and chromosomal loci encompassing genes for enhanced virulence and multiple drug resistance (Figure 2 Panel G). In brief, the cKp has the basic genetic 'makeup' with conserved genes represented by those encoding the enterobactin and fimbriae and it may evolve into the HvKp once genes encoding the unique siderophores and mucoid phenotypes appeared in the structure. Alternatively, it may also turn into the CR-Kp with acquisition of genes responsible for the extended-spectrum β-lactamase (ESBL) and carbapenemases (KPC). Conceivably, simultaneous possession of these genes yielded the CR-HvKp. In aggregation, this accessory genome and the strain-specific genes it harbored not only distinguished these Kp strains but made identification of the individual strain possible by using the mNGS technique. In general, the mNGS works by mapping the sequenced reads (sequences generated by a high-throughput sequencing platform) from the pathogen under investigation to an existing database of previously known microbes and aligning for bioinformatics analysis 21. As such, cKp strain could be identified precisely from a given human clinical sample and positive reads of the ESBL and KPC genes, such as *bla*_CTX-M_*, bla*_TEM_, *bla*_SHV_ and *bla*_KPC_, may further break the CR-Kp strain from the genetic background of cKp 22. Likewise, the hvKp strain could be singled out by detection of biomarkers including the *peg-344*, *iroB*, *iucA*, *rmpA* and *rmpA2* with high accuracy 23. Detailed descriptions of the mNGS were beyond the scope of our current work but available elsewhere 21-23. Differentiation of these strains may essentially link genetic profiles of an individual one to its distinctive clinical observations.

This scenario was best exemplified by isolation of the New Delhi metallobeta-lactamase 1 (NDM-1) in 2008 from a local Kp strain, which contained a transmissible genetic element encoding multiple drug resistance genes 24. The original organism was found to be resistant to all antimicrobial agents tested except colistin and the ability of plasmid to spread rapidly among *Enterobacteriaceae* strains subsequently led to the worldwide dissemination of the NDM-1, all of which brought our focus back to the β-lactamases. According to the Ambler classification, β-lactamases were categorized into class-A (ESBL and KPC), -B (metallo-β-lactamases, MBL), -C (cephalosporinases, AmpC) and -D (oxacillinases, OXA) 25. As a potent novel agent, ceftazidime avibactam sodium provides excellent coverage for the ESBL, KPC, AmpC and OXA-48. However, it is inefficient against the MBL and the addition of aztreonam may literally potentiate activity against CR-Kp that expressed both serine β-lactamases and MBLs (Figure 2 Panel H) 26. Arguably, a knowledge-based decision was the key to the salvation of critically ill patients especially accompanied by impaired renal function. To further highlight this notion, severe infection due to hvKp in which disseminated abscesses irreversibly victimized the AVF was delineated hereunder.

### Severe infection caused by the hvKp

**Figure 3:**
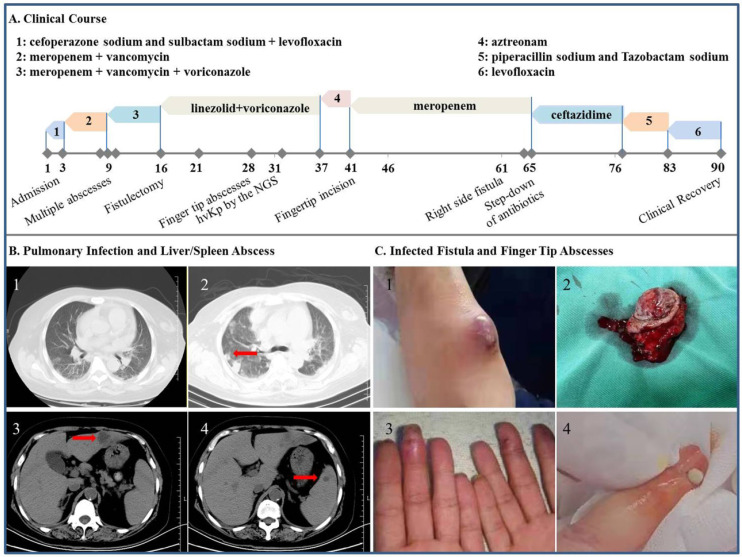
** Clinical, imaging and morphologic characterization in the patient with hvKp infection. A:** Clinical course of the patient's hospital stay. **B1:** Chest CT scan at admission. **B2-4:** CT scan on hospital day 9 showing abscess of the lung, liver and spleen (arrow), respectively. **C1 and C2:** Tumoral dilation and purulent exudation of the arteriovenous fistula, prior to and after the fistulectomy, respectively; **C3 and C4:** scene reconstruction of the fingertip abscesses.

A 55-year-old diabetic female on MHD with chills and fever of 38.0 ºC for 2 days was referred to us for further evaluation (Figure 3). She was given antipyretics but not antibiotic at local clinic and her fever only receded briefly. Further, tremor of her native AVF on the left forearm has disappeared 1 day prior to her arrival, which led to thrombolysis with urokinase and massage of the AVF at that time. On arrival, her AVF had no sign of inflammation and palpable tremor, whereas fingertips of her left hand were cyanosis. Laboratory tests showed leukocytosis, decreased hemoglobin concentration, platelet count and major elevation of inflammatory markers (Table).

As schematically depicted in Figure 3, the patient was given cefoperazone sodium sulbactam sodium and levofloxacin intravenously. Yet her fever continued unrelentingly and she further became delirious with platelet count plummeted to 16×10^9^/L on hospital day 3, at which time meropenem and vancomycin were started. The symptoms culminated at hospital day 5, exhibiting a temperature of 39.7 °C, type II respiratory failure and suspected bacterial embolus in the right atrium, followed by gangrene of the left fingertips on hospital day 7 and multiple abscesses of the lung, liver and spleen detected on day 9. The antibiotic regimen was further augmented with voriconazole. Nevertheless, her AVF progressively appeared to have tumoral dilation and purulent exudation, prompting fistulectomy on hospital day 16 which swiftly normalized her temperature and mitigated the inflammatory markers the following day (Table). The fingertip abscesses were handled surgically and mNGS of the removed tissue identified virulence genes (*iucA* and *rmpA*) peculiar to the hvKp and subsequently defined this strain, which was sensitive to the majority of antibiotics. Only after thorough cleanup of these sources of infection, was a *post-hoc* de-escalation of antibiotics possible and clinical recovery fulfilled. Of particular note is that endophthalmitis was ruled out by ophthalmologists and the patient maintained visual acuity during her hospital stay.

The hvKp was first recognized in Taiwan in 1986 and has undergone epidemic spread in Asia, especially South Korea, Japan and China, with sporadic but increasing rates reported elsewhere 27. For the most part, prevalence and lethality of the hvKp own to its enhanced pathogenicity and/or virulence. The most critical virulence factors characterized so far for the pathogenic hvKp are increased production of hypercapsule and siderophores 28. In fact, the latter are the bacteria-secreted proteins with a high affinity for iron, in which the aerobactin accounts for more than 90% in hvKp's total siderophore production. These two factors may respectively enable the hvKp strains extra protection against phagocytosis by the immune cells and iron acquisition needed for infection. Hence, unique *modus operandi* of the hvKp is responsible for its infection and dissemination among the population, irrespective of healthy and vulnerable ones.

The hvKp infection in our patient initially manifested as high fever and hypoxia, with a surge in the inflammatory markers and concomitant thrombocytopenia, which was an early manifestation and independent predictor of mortality in sepsis 29. Consistent with the invasive nature of hvKp, multiple abscesses developed in short term. Further, there were retrograde fingertip abscesses caused by massage of the AVF, which was somehow accepted as an expedient following thrombosis 30. Generally speaking, AVF is considered to have a low infectious risk with an estimated rate of 2-4% 31 and in most cases respond well to antibiotic treatment of 4-6 weeks. By stark contrast, AVF infection by the hvKp appeared to be much more serious as it was associated with aneurysmal enlargement and abscesses. Once infected, the AVF may turn out to be a cesspool of pathogens and is difficult to cure with a consequence of intermittent seeding of bacteria into the blood with septic reactions. As a lesson learnt, the infection in our case was not curtailed despite 'full-spectrum coverage' of antibiotics until the infected AVF was excised and fingertip abscesses drained. In aggregate, our report accentuated that dialysis staffs should be well versed in managing the hvKp infection, in which AVF involvement should be carefully evaluated during the bacteremia and its extrusion prohibited.

Positivity rate of blood culture for microorganisms including the cKp was found to range from 3.6 to 13.0% 32. The reason was attributed to the low propensity of pneumonia to give bacteremia or/and exposure to empirical antibiotic use. Fortunately, new technology such as the mNGS may help clinical microbiology laboratories acquire the capability to routinely identify diverse Kp strains. Beginning with prompt recognition, key managements of the hvKp infection included rapid initiation of therapy to prevent subsequent spread, detection of occult abscess to expedite source control, and appropriate site-specific therapy (e.g., meningitis, endophthalmitis, and prostatic abscess). From our own experience combating infectious diseases in patient with chronic kidney diseases and on MHD, the treating physicians need more keen attention to meningitis which may be mistaken for metabolism-related changes in mental status, endophthalmitis 16 or brain abscesses 33. And additional imaging (CT or magnetic resonance imaging) was recommended in search of latent sites of infection, which may be unrecognized and hamper source control (e.g., drainage).

### Detection of the CR-hvKp strain in critically ill patient

**Figure 4:**
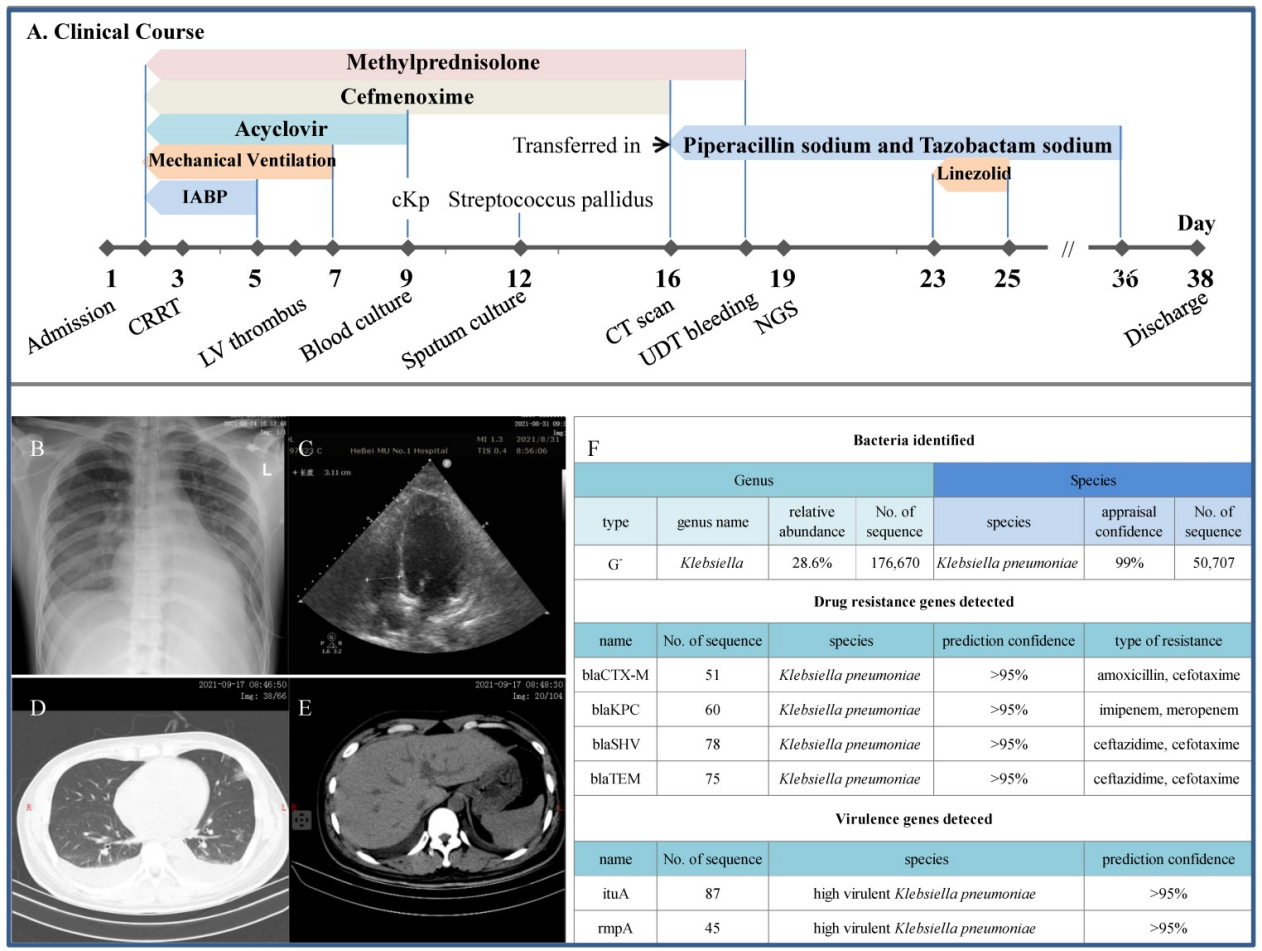
** Clinical course and imaging findings in the patient with positive isolation of CR-hvKp. A:** Major therapeutic procedures during the patient's hospital stay. **B:** Bedside plain X-ray showing cardiomegaly and pulmonary edema. **C:** Echocardiography showing left ventricular thrombus. **D and E:** CT scan at the day of transfer showing pulmonary patchy ground-glass opacity, pleural effusion and gross normal liver, respectively. IABP: intra-aortic balloon counterpulsation. **F:** mNGS results showing the detection of multiple drug resistance genes including the KPC and characteristic genes for hvKp.

A 28-year-old nondiabetic young man was rushed to the ICU for fever of 38.0-39.5 ºC and obvious dyspnea (Figure 4). He was given acyclovir and cefmenoxime coupled with methylprednisolone for the treatment of viral myocarditis and kept on intra-aortic balloon counterpulsation and mechanical ventilation from the second day for cardiogenic shock when bedside X-ray cogently revealed cardiomegaly and pulmonary edema. AKI also occurred shortly requiring CRRT and his condition was further complicated by left ventricular thrombus, positive blood culture for cKp and sputum culture for streptococcus pallidus. When transferred to us on the 16^th^ day, the patient was still receiving steroid and anticoagulants, and had intermittent fever and cardiac murmur. *A priori*, the antibiotic regimen was converted to piperacillin sodium and tazobactam sodium which was later reinforced by linezolid briefly. Most notably, mNGS technique did identify CR-hvKp from the sputum with its definite 'genetic fingerprints' of drug resistance and hypervirulence (the* bla*, *rmpA* and i*utA*). Despite an episode of upper digestive tract bleeding, the patient was safely discharged in the absence of all said abnormalities.

By comparison, this patient has probably been exposed to the highest risk of developing fatal sepsis. This speculation was based on the facts that he had cardiogenic shock, steroid therapy and CR-hvKp sprouted from the sputum. In this aspect, patients with kidney diseases receiving steroid were more susceptible to deadly infections caused by opportunistic pathogens as we have previously reported 16, 33, 34. It is thus quite likely that the sputum-bound CR-HvKp may end up in his blood stream and this close call was sufficient to ring the alarm. As a deeper thought, the incidence of CR-hvKp infection in dialysis unit was merely a matter of 'when' instead of 'whether' since the cKp strains are competent to change rapidly and acquire new traits. Congruous with our concern, outbreak of CR-hvKp in a Chinese hospital had inexorably wiped out all the infected 35. These findings implied the transmission of mobile genetic elements between hvKp and CR-Kp strains. More alarmingly, epidemiological analysis of recent hvKp clinical isolates in China indicated a potential for the global dissemination of hvKp strains with extensive antibiotic resistances in the near future 25. Indeed, prevalence of hvKp among CR-Kp isolates in China is presumably high, ranging from 7.4% to 15.0% (36). Therefore, professional awareness to the looming threat of hvKp with resistance determinants, especially against carbapenem, is greatly needed.

## Conclusion

The need to meet the challenge of evolving* Klebsiella pneumoniae* and its ultimate yield of CR-hvKp in the MHD patients is paramount. By summarizing present understanding of this deadly and variable pathogen in the said patient population, our work may undoubtably be of great help to dialysis staffs when fighting this devastating infection.

## Figures and Tables

**Table 1:** Clinical features and laboratory results during the patient's hospital stay

	Admission	Pre-fistulectomy	Post-fistulectomy	Finger Abscesses	post-Abscess incision
Hospital Stay (day)	1	14	17	36	44
T (ºC)	38.3	38.3	37.0	36.3	36.4
SaO2 (%)	88	97-99	97-99	97-99	97-99
BP (mmHg)	132/80	135/72	132/77	138/85	150/95
WBC (×10^9^)	15.0	14.8	13.6	6.1	6.1
Neutrophil (%)	96.3	84.6	87.7	75.1	64.0
Lymphocyte (%)	2.8	6.5	6.6	13.4	16.3
Hemoglubin (g/L)	80	56*	97	68	83
Platelet (×10^9^)	56	89	263	184	366
NLR	34.4	13.0	13.3	5.6	3.9
CRP (mg/L)	350.5	117.4	97.0	8.3	7.5
PCT (ng/mL)	>50.0	13.0	3.1	3.1	0.5
D-dimer (ng/mL)	1005	4830	5102	688	404
Albumin (g/L)	33.4	21.5	24.3	29.4	32.1
GOT (U/L)	87.3	13.0	18.3	14.5	19.1
GPT (U/L)	69.2	11.8	12.1	12.4	13.8
Scr (µmol/L)	778.8	795.8	649.4	600.8	404.1
FBS (mmol/L)	26.4	9.1	8.0	7.5	7.7
Ferritin (ng/mL)	992.7	1007.2	721.6	671.0	853.2

BP: blood pressure. WBC: white blood cell. NLR: neutrophil-to-lymphocyte ratio. CRP: C-reaction protein. PCT: precalcitoninnin; GOT and GPT: glutamate oxaloacetate transaminase and glutamate pyruvate transaminase, respecctively. Scr: serum creatinine; FBS: fasting blood sugar. *Blood transfusion initiated.
